# An Immediate Innate Immune Response Occurred In the Early Stage of *E*.*granulosus* Eggs Infection in Sheep: Evidence from Microarray Analysis

**DOI:** 10.1371/journal.pone.0135096

**Published:** 2015-08-07

**Authors:** Wenqiao Hui, Song Jiang, Jishun Tang, Hongyan Hou, Sheng Chen, Bin Jia, Qian Ban

**Affiliations:** 1 Institute of Animal Husbandry and Veterinary Medicine, Anhui Academy of Agriculture Sciences, Road Nongkenan, Hefei, 230031, Anhui, People’s Republic of China; 2 College of Animal Science and Technology, Shihezi University, Road Beisi, Shihezi 832003, Xinjiang, People’s Republic of China; 3 Center for Stem Cell and Translational Medicine, School of Life Sciences, Anhui University, Road Jiulong, Hefei, 230000, Anhui, People’s Republic of China; Public Health Research Institute at RBHS, UNITED STATES

## Abstract

**Background:**

Cystic Echinococcosis(CE), caused by infection with the larval stage of the cestode *Echinococcus granulosus* (*E*. *granulosus*), is a chronic parasitic zoonosis, with highly susceptible infection in sheep. However, the comprehensive molecular mechanisms that underlie the process of *E*. *granulosus* infection in the early stage remain largely unknown. The objective of this present study was to gain a cluster of genes expression profiles in the intestine tissue of sheep infected with CE.

**Methods:**

Nine healthy sheep were divided into infection group and healthy controls, with six infected perorally 5000 *E*. *granulosus* eggs suspended in 1000μl physiological saline and three controls perorally injected 1000μl physiological saline. All animals were sacrificed at 4 hours post-infection, respectively. The intestine tissue was removed and the RNA was extracted. In the infection group, the biology replicates were designed to make sure the accuracy of the data. The ovine microarrays were used to analyze changes of gene expression in the intestine tissue between CE infected sheep and healthy controls. Real-time PCR was used to assess reliability of the microarray data.

**Results:**

By biology repeats, a total of 195 differentially expressed genes were identified between infected group and controls at 4 hours post-infection, with 105 genes related to immune responses, while 90 genes associated with functions including energy metabolism, fat soluble transport, etc. Among the 105 immunity genes, 72 genes showed up-regulated expression levels while 33 showed down-regulation levels. Function analysis showed that most of up-regulated genes were related to innate immune responses, such as mast cell, NK cell, cytokines, chemokines and complement. In addition, Real-time PCR analysis of a random selection of nine genes confirmed the reliability of the microarray data.

**Conclusion:**

To our knowledge, this is the first report describing gene expression profiles in the intestine tissue of CE infection sheep. These results suggested that the innate immune system was activated to elicit immediate defense in the intestine tissue where *E*. *granulosus* invaded in at 4 hour-post infection. Furthermore, future interest will also focus on unraveling similar events, especially for the function of adaptive immunity, but at late stage infection.

## Introduction

Cystic echinococcosis (CE) is a chronic parasitic zoonosis caused by infection with the larval stage of the cestode *Echinococcus granulosus* (*E*. *granulosus*), resulting in the development of cysts in human and domestic animals, especially in sheep, which appear to be highly susceptible to infection [1]. After oral uptake of *E*. *granulosus* eggs by host, the activated oncosphere, which hatched by *E*. *granulosus* eggs in the gastrointestinal tract, penetrate the intestinal wall and subsequently enter the bloodstream, eventually locating in viceral organs, such as liver, lung, heart and kidney, where they develop into hydatid cysts. Characterized by long-term growth of the metacestode (hydatid) cysts in internal organs, CE not only presents a substantial threat to human health in areas of poor sanitary and hygiene, but also causes serious animal health problems in many rural areas of the world, resulting in economical losses due to viscera condemnation and decreased productivity, such as reductions of live weight gain, low rates of fertility, and poor value of wool [2, 3]. Hence, CE control is a vital part of health and production management in domestic animals, especially in sheep, as sheep are the major intermediate ruminant hosts of CE [4]. As one aspect of CE controlling program, immunology and immunodiagnosis studies have received much more attention in the past two decades [4, 5], because understanding how the immune responses are produced is extremely important in developing immunodiagnostic kits and highly effective recombinant vaccines against *E*.*granulosus* infection. It has been shown that *E*.*granulosus* infection induced numerous pathways of the immune response. Researchers found that a marked activation of cell-mediated immunity was observed during the early stages of echinococcosis [6]. In addition, it has been demonstrated that Th1 cell activation, through cytokines IFN-gama, IL-2, IL-12, has the effective beneficial implication in protect immunity of echinococcosis [7], while Th2-dominated immunity was suggested to be associated with the susceptibility of the disease [8, 9]. However, much useful information is obtained from human, the molecular immunity of *E*. *granulosus* infection in livestock, e.g. sheep, is still poorly understood.

The intestinal mucosal immune system is the first line of defense against microbial, virus and parasite infection in the intestine tissue, which plays a vital role in maintaining intestinal homeostasis. The multiple host intestinal mucosal immunity includes the epithelial production of protective mucin, epithelial cell secretion of a broad range of antimicrobial peptides, as well as the involvement of dendritic cells, lymphocytes and related cytokines [10].

As an orally acquired pathogen, the immune response to *E*. *granulosus* should logically unfold in the small intestinal mucosa. Previous study carried out in experimentally *E*. *multilocularis* infected mice has shown that intestinal immunity plays a pivotal role in stopping the larval growth of *E*. *multilocularis* at the very early stages [11]. Nevertheless, the comprehensive molecular mechanisms that underlie the process of *E*. *granulosus* infection in the intestine tissue remain largely unknown, especially in sheep, although previous studies carried by us showed that sheep with different MHC haplotype could produce a significant system immune response against *E*. *granulosus* infection, such as antibody and Th1/Th2 cytokines [12–14].

High-throughput methods, e.g. DNA microarrays, can provide a comprehensive picture of the genes underlying the host responses to disease. Recently, several studies have been performed in mice to detect differently expressed genes in the liver tissue during the process of alveolar echinococcosis (AE) infection, one kind of echinococcosis [15–17]. However, to our knowledge, few studies have been reported on transcriptional profiling in the intestine tissue after echinococcosis infection. It is, therefore, necessary to conduct a genome-wide characterization of genes during *E*. *granulosus* infection in the intestine tissue stage.

In sheep, more and more transcriptomic studies were carried out using microarray analysis in the past decades [18–24]. Some researchers have used microarray technology to attempt to describe genome-wide expression differences in host responses to diseases, including gastronintestinal nematodiasis [18–21], prion diseases [22], and even the disease model like congenital heart disease [24]. However, so far, few reports on gene expression-profiling changes in the intestine tissue of sheep with CE were published.

To gain a comprehensive view of the CE transcriptional landscape during the early stages of infection, we used microarrays to analyze changes in gene expression in a sheep model. In the present study, an ovine microarray technology was applied by us, to identify transcriptional changes in the intestine tissue of sheep that were primary infection with *E*. *granulosus*, which may help understand the molecular and immunology mechanism that underlie the process of *E*. *granulosus* infection in the early stage.

## Materials and Methods

### Ethics statement

All animals were raised and handled in strict accordance with the Animal Ethics Procedures and Guidelines of the People’s Republic of China. The protocol was approved by the Institutional Animal Care and Use Committee of Shihezi University.

### Animals and experimental design

A total of nine one year old healthy Kazakh sheep were raised in parasite-free conditions. They were divided into infection and control groups, with six sheep in infection group and three individuals in the control group. Prior to experimentally infected with *E*. *granulosus* eggs, all of sheep were negative for antibodies to hydatid cyst fluid (HCF) antigen, assayed by a commercial ovine hydatidosis ELISA kit (Shenzhen Combined Biotech Co., Shenzhen, China), and no hydatid cysts presented in internal organs detected by ultrasonography (50S Tringa VetPie Medical, Netherland).

*E*. *granulosus* eggs were obtained from the gut content of a highly-infected dog according to the method reported by Zhu et al [25]. Strict safety procedures were followed during the handling of eggs [26]. In the infection group, each sheep was infected perorally via a medical syringe with 5000 *E*. *granulosus* eggs suspended in 1000μl physiological saline. Another three individuals selected as healthy controls were perorally injected 1000μl physiological saline. At 4 hours post-infection, animals were sacrificed by administration of the euthanasia medicine containing hydroxybutyramide, methylene ammonium iodide, tetracaine (100 mg/kg, IV route). Immediately afterwards, the intestine tissue was removed and approximately 10 mm^3^-sized intestine tissue blocks were collected and frozen immediately in liquid nitrogen prior to long-term storage at -80°C until RNA extraction.

In terms of infection group, we designed biology replicates: the RNA of three sheep were mixed as RNA pool, and named as group A, while RNA of the other three counterparts were mixed as its biology replicates, named group B. Likewise, the RNA of three controls were mixed and named as group C.

### Sheep oligo-DNA microarray

The chip used for the test was a sheep whole-genome microarray (4×44k microarrays) provided by Beijing Protein Innovation Company, which contained 15,008 probes.

### Labeled cRNA preparation and Microarray hybridization

The first strand cDNA was synthesized, followed by second strand cDNA synthesis. cDNA was then transcribed and labeled with T7 RNA Polymerase and cyanine 3-CTP. After purified by RNeasy Mini Kit (Qiagen), the labeled cRNA was fragmented to segments using fragmentation buffer at 60°C for 30 min. After fragmentation, the samples were then hybridized to Agilent custom 4×44k chips. The hybrid was carried out in a rolling hybridization oven for 17 hours at 65°C and 6 rpm. The completed hybridization slide was removed from the oven, and was washed using Gene Expression Wash Buffer Kit (Agilent), according the manufacture’s instruction, and then scanned by Agilent Microarray Scanner. Hybridizations of each sample were performed in duplicate. All microarray procedures were done at Beijing Protein Innovation Co., Ltd.

### Real-time PCR validation

Real-time PCR was employed to verify the regulation of genes detected by microarray. Nine differential expression genes were randomly selected as quantitative real-time PCR analysis. The GAPDH was used as an internal control. The primers for these genes, listed in Table 1, were designed by Primer premier 5.0 and synthesized by Beijing BGI Company. Real-time PCR was performed under the following condition: an initial denaturing step at 94°C for 4min, followed by 35 cycles of 94°C for 15s, annealing at 49–59°C for 20s, and extension at 72°C for 20s, and a final extension step of 10 min at 72°C. Each reaction was carried out in a total volume of 20μl, consisting of 12.5μl SYBR Premix Ex, 0.5μl each primer (10μ mol/L), 2μl cDNA and 4.5μl ddH_2_O. Amplification reactions in triplicate for each sample were performed. And the levels of transcript were generated from a standard that was simultaneously amplified with the samples. Levels of gene expression were then normalized against GAPDH, which served as internal controls.

**Table 1:** Primer sequence for qRT-PCR analysis of gene transcripts.

Gene	Primer sequences (5’-3’)	Annealing temperature (°C)	Expected size (bp)
GAPDH	F: CTGACCTGCCGCCTGGAGAAA	59.0	149
	R: GTAGAAGAGTGAGTGTCGCTGTT		
MCP1	F: AGCCTCACTCCCGTCCCTA	55.0	283
	R: TCCGACATCTCAGCCTTCCTA		
KLRJ1	F: TGAATGGAAGTGGGAGGAT	50.0	141
	R: CACGAGGAAGACATGGTGC		
CII-TA	F: CAAAGCATGACCGCTGGAAATT	57.0	209
	R: AAACAAACAGGAAATGGAGGCAAA		
CXCL17	F: CAGGCTTCTGGGAGGTGGCT	55.0	109
	R: CTCTGGAGGGCTTGGTTGG		
ITLN2	F: CTCTGTGCTGAGCCCGAGAC	55.0	236
	R: CATCACCTGCTTTACGACATCTTT		
FCER1A	F: AGATGGGACATTGTGAAAGC	49.0	167
	R: CACCTGAGGAAGAGGGACT		
SLC2A5	F: AGTGAATGCGATCTACTACTACGC	53.0	196
	R: GCAGCAGGCAGTGAAACAG		
CXCR6	F: ATCTTGGCGTCTTAGACCTTCAT	55.0	180
	R: TCTTCAGCCGACCCTGTTTT		
TNFR1	F: GCAGGAAGAACCAGTACCGGGAATA	59.0	221
	R: AGTTGAAGGTCGGGTTGGACATAA		

### Data analysis

The hybridization data were extracted with Feature Extraction Software 10.7. Gene Spring 12.0 Software was used for data analysis. The read dates were normalized, and only genes with a Student’s t test *P*-value<0.05 and a fold change(FC)≥2 were selected as differential expression ones.

## Results

In this study, changes of the sheep intestine gene expression in response to primary *E*. *granulosus* infection were examined during the very early stage of infection. Actually, *E*. *granulosus* infection resulted in a wide range of gene expression in sheep. After 4h *E*. *granulosus* infection, 291 differentially expressed genes were identified between group A and group C (FC≥2, P<0.05). For its biology repeats, there were 274 differentially expressed genes between group B and group C. Interestingly, a total of 195 genes, occurred both in the two comparisons, with 105 genes were related to immune responses, while 90 genes associated with functions including energy metabolism, fat soluble transport, etc. By biology repeats analysis, these 195 differential expression genes were screened as genes associated with CE infection in the intestine tissue of sheep. Among them, 72 genes showed up-regulated expression levels while 33 displayed down-regulation levels. Most of up-regulated genes were related to innate immune responses, including innate immunity cells (macrophages, NK cell, mast cell, basophils) and innate immunity molecules (cytokines, chemokines, complement, lysozyme, intelectin 2). Table 2 listed the information of partially differential expression genes detected in the present study.

**Table 2:** Partial differential expression genes in the intestine, organized according to their function groups.

Gene	Accession number	Gene ID	Description	Ratio	
				A vC	BvC
**Up-regulated genes Genes associated with gut mucosal epithelial tissue immunity**					
Lysozyme C2	NM_180999.1	30794293	lysozyme C-2	27.0	28.1
ITLN2	NM_001104934.1	100125357	intelectin 2	92.0	93.5
**Genes associated with innate immunity cell**					
Nature killer cells (NK cell)					
KIR2DS1	NM_001097567.1	493737	killer cell immunoglobulin-like receptor, two domains, short cytoplasmic tail, 1	5.1	2.4
KLRJ1	NM_001002884.1	444861	killer cell lectin-like receptor family J member 1	16.1	9.6
MPTX	NM_001034227.1	504879	putative mucosal pentraxin homolog (MPTX), mRNA	11.7	13.0
Mast cell					
MCP3	NM_001009411.1	443429	mast cell proteinase-3	43.1	31.4
MCP1	NM_001009472.1	443546	mast cell proteinase-1	42.4	31.1
IgE Fc receptor	NM_001123004.1	100144427	Fc fragment of IgE, high affinity I, receptor for; alpha polypeptide	24.7	37.7
IRAK4	NM_001142514.1	100216438	interleukin-1 receptor-associated kinase 4	2.7	2.7
Macrophages					
IL-6	NM_001009392.1	443406	interleukin 6	4.9	5.0
Innate-like lymphocytes (ILLS):B1 cell					
IgM	AY145128.1	24496448	immunoglobulin M heavy chain	10.2	12.4
**Genes associated with innate immunity molecule**					
Cytokines and Chemokines					
Th1 cytokines and related chemokines					
IL-2	NM_001009806.1	443401	interleukin 2	2.7	2.6
IL-3	NM_001009420.2	443438	Ovisaries interleukin 3 (colony-stimulating factor, multiple) (IL3), mRNA	3.4	3.8
TNF13 B	NM_001114506.1	504507	tumor necrosis factor (ligand) superfamily, member 13b	2.3	3.4
CXCL2	NM_001046513.1	613667	chemokine (C-X-C motif) ligand 2	2.8	4.7
CXCL5	NM_174300.2	281735	chemokine (C-X-C motif) ligand 5	2.8	5.2
CXCL9	NM_001113172.1	513990	chemokine (C-X-C motif) ligand 9	2.0	2.0
CXCL17	NM_001083799.1	788717	chemokine (C-X-C motif) ligand 17	4.2	4.7
CCL28	NM_001129903.1	780500	chemokine (C-C motif) ligand 28	3.2	2.4
Th2 cytokines and related chemokines					
IL-13	NM_001082594.1	664707	interleukin 13	3.8	5.2
CCR4	NM_001100293.1	408019	chemokine (C-C motif) receptor 4	2.8	4.0
CCR3	NM_001009241.1	443111	chemokine (C-C motif) receptor 3	2.7	3.1
Complement					
C4BP	XM_002693873.1	515150	complement component 4 binding protein, alpha chain-like (LOC515150), mRNA	7.7	8.0
CFHR5 (C8BP)	XM_514078.2	457605	complement factor H-related 5 (CFHR5), mRNA	3.8	7.2
**Genes associated with adaptive immunity**					
CIITA	XM_858458.1	73958939	MHC class II transactivator, transcript variant 3	8.9	5.5
**Other up-regulated genes associated with non-immunity**					
SLC5A6	NM_001177622.1	330064	solute carrier family 5 (sodium-dependent vitamin transporter), member 6	223	102.1
TM4SF4	NM_001034306.1	507646	transmembrane 4 L six family member 4	58.6	53.5
SLC2A5	NM_001009451.1	443507	solute carrier family 2 (facilitated glucose/fructose transporter), member 5	34.0	48.8
UGT2A3	NM_001098944.1	511743	UDP glucuronosyltransferase 2 family, polypeptide A3	26.6	25.8
PDE6C	NM_174419.1	281975	phosphodiesterase 6C, cGMP-specific, cone, alpha prime	21.1	19.4
**Down-regulated genes**					
CXCR6	NM_001014859.1	506807	chemokine (C-X-C motif) receptor 6	2.3	2.4
CXCL1	XM_002694885.1	517354	chemokine (C-X-C motif) ligand 1	4.7	4.6
TNFR21	NM_001076911.1	537922	tumor necrosis factor receptor superfamily, member 21	2.7	3.8
TNFR1	NM_001166185.1	100135698	tumor necrosis factor receptor superfamily, member 1A	2.8	2.5
C1QTNF6	NM_001101872.1	506413	C1q and tumor necrosis factor related protein 6	2.5	3.5

Note: Table 2 shows partially differential expression genes (*E*. *granulosus* challenged at four hour *vs*. Healthy controls) with *P*-value<0.05 and fold change (FC)≥2, which were listed with their respective function.

The most prominent genes which were exceedingly up-regulated in the intestine tissue at 4 hour post infection with *E*. *granulosus* encode intelectin 2 (ITLN2) and lysozyme C2, responsible for the first line of host defense against parasite (Table 2). Consistent with the up-regulation of above antimicrobial peptides secreted by the intestine tissue, the represented up-regulated genes involved in the innate cell immunity are MCP1, MCP3, FcεRI and IRAK-4, responsible for the activation of mast cell pathway; KIR2DS1, KLRJ1 and MPTX, mainly associated with NK cells; as well as IL-6 secreted by Macrophages and IgM predominantly produced by B1 cells (Table 2). Besides, genes associated with innate molecular immunity were also up-regulated in the intestine tissue at 4 hour post infection with *E*. *granulosus*, which encode Th1 cytokines (IL2, IL3, TNF13 B) and related chemokines(CXCL2, CXCL5, CXCL9, CXCL17, CCL28), as well as Th2 cytokiens and related chemokines (IL13, CCR3, CCR4). Furthermore, genes encoding complement pathway factors (C4BP, CFHR5) were significantly up-regulated at 4 hour post infection with *E*. *granulosus*. It’s worth noting that CIITA, the major histocompatibility class II (MHCII) transactivator (CIITA), which specifically regulates MHCII expression, manifested a significantly higher expression level in CE infection sheep at four hour post infection (Table 2).

In addition to immunity, genes associated with metabolism (e.g. SLC5A6, SLC2A5, UGT2A3, and PDE6C) as well as membrane proteins, like TM4SF4, were also detected with a significantly higher expression level in CE infection sheep at four hour post infection, when compared with healthy controls (Table 2).

**Fig 1:**
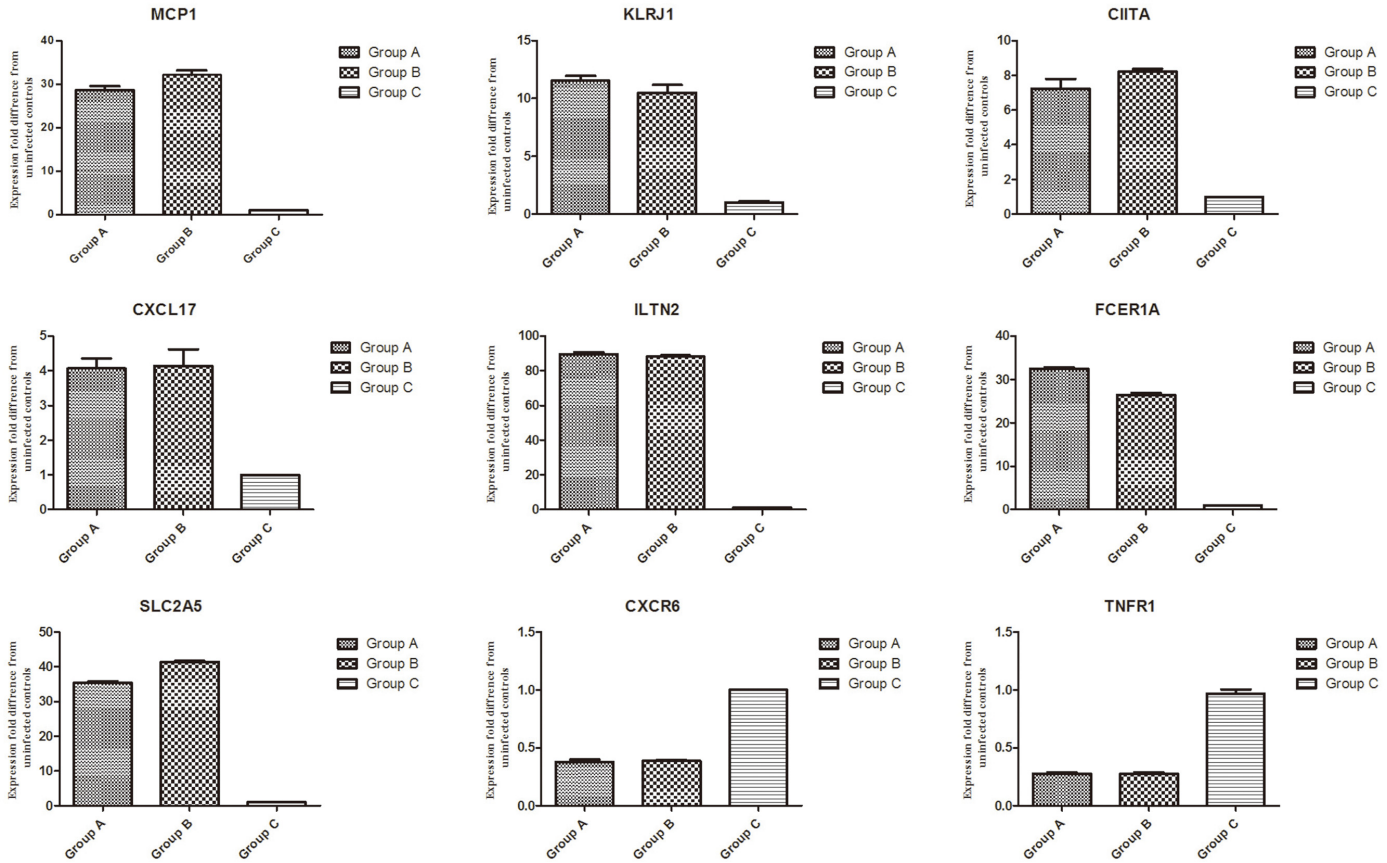
Validation of microarray data by qPCR on 9 randomly selected genes. Real-time PCR was employed to verify the regulation of genes detected by microarray. Nine differential expression genes were randomly selected as quantitative real-time PCR analysis. The GAPDH was used as an internal control.

Nine genes with a differential expression level were randomly selected to conduct Real-time PCR validation of microarray data. The results from Real-time PCR were shown in Fig 1, which were highly correlated with those generated from microarray analysis.

## Discussion

Studies regarding early and local immunity responses in primarily *E*. *granulosus* infection of sheep are virtually absent from current literature. Thus, in order to better understand the molecular events characterizing the host response to CE at the very early stage, we used microarrays to analyze changes of gene expression at 4h post infection in sheep. Four-hour post infection was selected partially due to our prior finding that the maximal IgE and Th1 cytokines was detected in serum at 4h of *E*. *granulosus* infection in CE resistance sheep [7]. Moreover, it has been demonstrated that the period of 0-4h post-infection is hosted by immediate innate immunity, including the barrier function of mucosal, the activation of cells immunity, as well as the production of proinflammatory cytokines and chemokines [27]. Therefore, we hypothesized that critical host immune mechanisms of very early responses to *E*. *granulosus* infection are active at this time.

**Fig 2:**
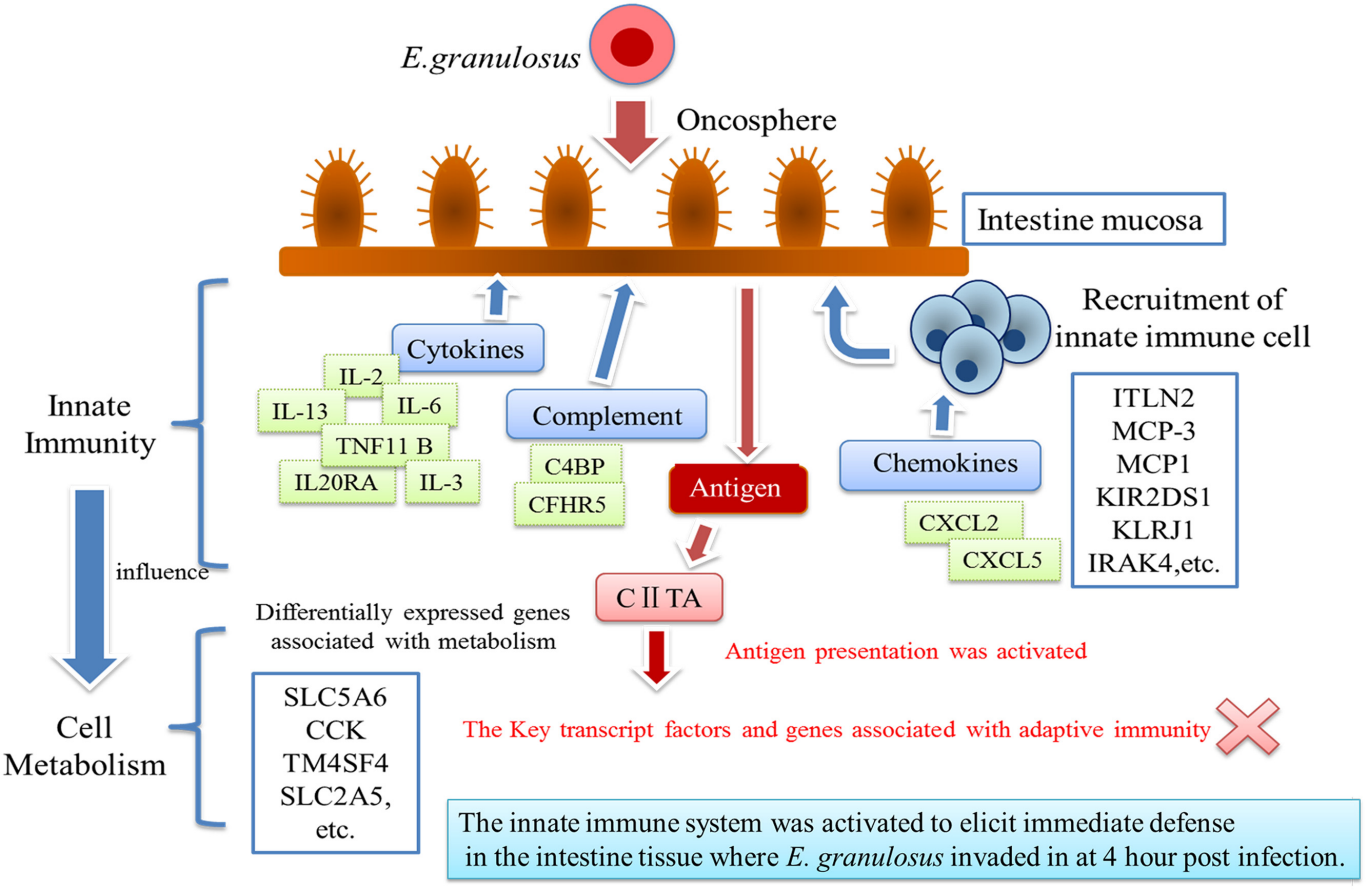
Schematic diagram summarizing interaction linked to differential expression genes in the intestine tissue of sheep infected with CE. Interaction of immune cell/molecular in the intestine tissue of sheep infected with CE as suggested by the pattern of differential expression genes listed in Table 2.

Actually, *E*. *granulosus* infection resulted in a wide range of gene expression at 4 hour post infection in sheep in the present study. Interestingly, most of up-regulated genes were related to innate immune responses, including cellular immunity and innate immunity molecular, such as cytokines, chemokines and complement. Our results pose a possible explanation for the early and intestinal events in *E*. *granulosus* experimental infection—the innate immunity was strongly activated by *E*. *granulosus* infection at 4 hour post infection, supported by Fig 2, which demonstrated the schematic interaction linked to differential expression genes.

Firstly, the epithelial layer of intestine tissue may play a physiological barrier at four-hour of *E*. *granulosus* infection.

The epithelial layer could produce a variety of antimicrobial peptides, including inlectin, lysozyme, defensin, etc., as well as mucus, thus providing a spatial separation of bacteria from the cell surface [10]. In the present study, genes associated with the intestinal epithelial cells (e.g. intelectin 2, lysozyme C-2) were significantly up-regulated in CE infection sheep, compared with healthy controls.

Previous studies have shown that the intelectin 2 plays an important role in expel intestine parasite infection [28], and the lysozyme participates in host defense against bacterial infection [29]. In this study, the above listed genes showed dramatically higher expression levels, suggesting that the gut mucosal immune system was strongly activated and response to *E*. *granulosus* infection at four hour.

Secondly, we found that innate immunity cells, including NK cells, mast cell, and macrophage, contribute to the immunological response and defense of *E*. *granulosus* infection at four-hour post infection. With regard to the intestine gene expression profiles of our study related to immune response, most of the highest expressed genes (KIR2DS1, KLRJ1, MCP1, MCP3, FcεRI, IgM) appear to be associated with innate immunity cells.

As a major component of the innate immune system, natural killer cells are responsible for activating the cytolytic killing of foreign pathogen infection, primarily in defenses against virus, bacteria and parasite during early infection [30]. Here, we have shown that genes encoding the activator of NK cells (KIR2DS1, KLRJ1) were markedly up-regulated in the intestine tissue of CE infection sheep at 4-hour post infection, suggesting that NK cells might be strongly activated, and thus pose a high cytotoxic potential very early during infection, which is supported by the high expression of MPTX gene encoding mucosal pentraxin. The pentraxin is a kind of anti-media released by NK cells, which is reported to be as clinical parameter in areas where parasite infections are [24]. Our results are in partial agreement with reports on sheep *C*. *parvum* infection, showing the highly activation of NK cells in the intestine tissue during the very early stage [30]. Although *E*. *granulosus* and *C*. *parvum* infections are dissimilar systems at the parasitological and immunological levels, such a common behavior in NK cells suggested that they participated in the first steps of the innate immune response, thereby playing important roles in the parasite infection at the very early stage.

In addition, the activation of FcεRI-dependent mast cell via TLR4 recognition plays an important role in the *E*. *granulosus* infection at 4-hour post infection, as indicated by the hyperexpressed molecules, such as FcεRI, MCP1, MCP3, and IRAK-4. Mast cells are immune cells that reside in almost all tissues and are most well known for their role in IgE- mediated responses. It has been demonstrated that mast cells are activated through cross-linking of the high-affinity IgE receptor (FcεRI). Besides, mast cells are mainly present in the strategic locations, where they may have a role in defense against parasites (mainly intestinal helminth parasites) and bacteria. These pathogens can be recognized by mast cell via Toll-like receptors [31]. In the present study, at four-hour of *E*. *granulosus* infection, FcεRI, MCP1, MCP3, and IRAK-4 were highly expressed in the intestine tissue of CE sheep, when compared with healthy controls. MCP1 and MCP3 encode mast cell proteases, which could active mast cells [32–34]. IRAK-4 is a mediator of TLR4 pathways, with an essential role in transducing downstream signals. Research work published showed that bacteria and viruses can influence FcεRI-dependent mast cell activation by influencing the surface expression of FcεRI [35]. It has been reported that both IgE and TLR4 could greatly enhanced the activation of mast cells, thereby producing inflammatory cytokines [31]. In the present study, the above listed phenomena putatively suggested the activation of FcεRI-dependent mast cell via TLR4 recognition plays an important role in the *E*. *granulosus* infection at 4-hour post infection.

In addition to NK cells and mast cells, macrophage may play important roles in Echinococcosis infection. Macrophages are the most abundant mononuclear phagocytes in the steady-state gut lamina propria [36]. In this study, IL-6, secreted by macrophages, involved in indirect macrophage pathways.

The above listed phenomenon indicated the important role of mast cells, NK cells an macrophages in Echinococcosis infection, especially for the eventual involvement of NO, knowing that its synthesis is highly induced by macrophages and up regulated by IFN-gamma and TNF-alpha, IL-6 [7].

B1 cells produce natural immunoglobulin M (IgM) antibodies that confer protection against *E*. *granulosus* infection. Studies have demonstrated that early IgM could protect against infection with viruses infection, such as influenza [37]. In this study, high expression of IgM was detected in CE infection sheep at 4 hour post infection, suggesting that B1 cell-derived IgM may play a certain role in defense against *E*. *granulosus* infection.

Thirdly, effective molecules in the innate immunity, including cytokines, chemokines and complement strongly contribute to the defense process of *E*. *granulosus* infection.

Cytokines are a kind of important regulatory and effect molecules participating in innate immunity. It has been reported that Th1 cytokines was associated with protective immunity in Echinococcosis, while Th2 cytokines was suggested to induce susceptibility of the disease [7–9]. Chemokines are considered as key regulators of leukocyte migration, which play important roles in a number of physiological and pathological immune and inflammatory processes [38]. In our study, we observed differential abundance of Th1 cytokines and related pathways, with most up-regulated (IL-2, IL-3, TNF13B), in the intestine tissue of CE infected sheep, accompanied by several over-expressed Th1 chemokines (CXCL2, CXCL5, CXCL9, CXCL17, CCL28). With respect to the intestine gene expression profiles of Th2–responses, only IL-13 cytokines, CCR3 and CCR4 chemokines were detected highly expressed in the intestine tissue of CE sheep. Globally, the above listed phenomena correspond mostly to a Th1-oriented immune response, which may putatively be the correct way to control infection. Besides Th1 cytokines and related chemokines, complement may contribute to the immunological and defense, as indicated by hyper-expressed CFHR5 and C4BP, which (Table 2).

Last but not least, the antigen processing system begins switch on after *E*. *granulosus*, which is suggested by the high expression of the major histocompatibility class II (MHCII) transactivator (CIITA), a non-DNA-binding coactivator that specifically regulates MHCII expression, being critical for normal immune function. In this study, CIITA expression in both infection groups up-regulated, compared with the healthy controls (Table 2). It is speculated that this phenomenon may in part be the result that *E*. *granulosus* infection stimulates the expression of CIITA gene, thereby active MHCII expression, which presents the antigen to the T cells. As factors that activate or inhibit MHCII expression act via the promoters that drive transcription of the MHCIITA gene [39]. The adaptive immune response is often required for complete control of infection [40]. In this study, the early increase of MHCIITA that we observed in the infection sheep probably coincides with the onset of adaptive immune response. As the period of 4h infection is too short to induce the response of genes in the adaptive immune system, to carry out a more profound interpretation of the present findings, we have to take account that the time point of infection status corresponds to the late stage.

## Conclusion

In order to gain a comprehensive view of the CE transcriptional landscape during the early stages of infection in sheep, the ovine microarray was applied by us to identify transcriptional changes in the intestine tissue of sheep that were primary infection with *E*. *granulosus*. Results showed that most up-regulated genes were associated with innate immune response: including gut mucosal immune; cells of the innate immune system, such as mast cell, nature killer cell, macrophage and even Paneth cell; as well as innate immune molecular, including cytokines, chemokines and complement. To our knowledge, this is the first report concerning gene expression profile in the intestine tissue of CE infection sheep. The data presented herein may suggest that the innate immune system was activated at four-hour post infection to elicit immediate defense in the intestine tissue where *E*. *granulosus* invaded in. Moreover, further interest will also focus on unraveling similar events, especially for the function of adaptive immunity, but at late stage infection.
